# The impact of a referral card-based intervention on intimate partner violence, psychosocial health, help-seeking and safety behaviour during pregnancy and postpartum: a randomized controlled trial

**DOI:** 10.1186/s12884-017-1519-x

**Published:** 2017-10-06

**Authors:** An-Sofie Van Parys, Ellen Deschepper, Kristien Roelens, Marleen Temmerman, Hans Verstraelen

**Affiliations:** 1 Department of Obstetrics and Gynaecology, International Centre for Reproductive Health, Ghent University, Faculty of Medicine and Health Sciences, De Pintelaan 185, UZP 114, 9000 Ghent, Belgium; 2 Department of Public Health, Biostatistics Unit, Ghent University, Faculty of Medicine and Health Sciences, De Pintelaan 185, 3K3, 9000 Ghent, Belgium; 3 Department of Obstetrics and Gynaecology, International Centre for Reproductive Health, Ghent University, Faculty of Medicine and Health Sciences, De Pintelaan 185, P4, 9000 Ghent, Belgium

**Keywords:** Intimate partner violence, Pregnancy, Intervention, Psychosocial health, Help-seeking behaviour, Safety behaviour

## Abstract

**Background:**

We aimed to investigate the impact of a referral-based intervention in a prospective cohort of women disclosing intimate partner violence (IPV) on the prevalence of violence, and associated outcomes psychosocial health, help-seeking and safety behaviour during and after pregnancy.

**Methods:**

Women seeking antenatal care in eleven Belgian hospitals were consecutively invited from June 2010 to October 2012, to participate in a single-blind randomized controlled trial (RCT) and handed a questionnaire. Participants willing to be interviewed and reporting IPV victimisation were randomised. In the Intervention Group (IG) participants received a referral card with contact details of services providing assistance and tips to increase safety behaviour. Participants in the Control Group (CG) received a “thank you” card. Follow-up data were obtained through telephone interview at an average of 10 months after receipt of the card.

**Results:**

At follow-up (*n* = 189), 66.7% (*n* = 126) of the participants reported IPV victimisation. Over the study-period, the prevalence of IPV victimisation decreased by 31.4% (*P* < 0.001), psychosocial health increased significantly (5.4/140, *P* < 0.001), 23.8% (*n* = 46/193) of the women sought formal help, 70.5% (*n* = 136/193) sought informal help, and 31.3% (*n* = 60/192) took at least one safety measure. We observed no statistically significant differences between the IG and CG, however. Adjusted for psychosocial health at baseline, the perceived helpfulness of the referral card seemed to be larger in the IG. Both the questionnaire and the interview were perceived to be significantly more helpful than the referral card itself (*P* < 0.001).

**Conclusions:**

Asking questions can be helpful even for types of IPV of low severity, although simply distributing a referral card may not qualify as the ideal intervention. Future interventions should be multifaceted, delineate different types of violence, controlling for measurement reactivity and designing a tailored intervention programme adjusted to the specific needs of couples experiencing IPV.

**Trial registration:**

The trial was registered with the U.S. National Institutes of Health ClinicalTrials.gov registry on July 6, 2010 under identifier NCT01158690).

**Electronic supplementary material:**

The online version of this article (10.1186/s12884-017-1519-x) contains supplementary material, which is available to authorized users.

## Background

Intimate partner violence (IPV) has been increasingly recognised as a worldwide health problem with serious clinical and societal repercussions that affect men and women from all backgrounds, regardless of socio-economic status, age, sexual orientation, religion or ethnicity [1–4]). IPV is defined as any behaviour in a present or former intimate relationship that leads to physical, sexual or psychological harm, including acts of physical aggression, sexual coercion, psychological abuse and controlling behaviour patterns [5]. Drawing upon the IPV definition provided by Saltzman [6], we have chosen to use consistently the term ‘violence’ for physical and sexual types of violence, and ‘abuse’ for psychological types since the word ‘abuse’ clearly implies a broader range of behaviours compared to ‘violence’, which is often associated with the most severe forms of violent behaviour. To avoid confusion in this paper, we will consistently use the term ‘IPVv’ (Intimate Partner Violence victimisation), ‘IPVp’ (Intimate Partner Violence perpetration) and IPV (both victimisation and perpetration) to refer to the specific behaviour measured in our sample. We are aware that these terms unavoidably hold normative connotations. Yet, to the best of our knowledge, accurate and more objective terms are currently unavailable.

The transition to parenthood brings joy as well as new challenges to intimate relationships [7, 8]. Pregnancy may be an exceptionally stressful time because of the multitude of changes in physical, emotional, social and economic roles and needs. Research has demonstrated that coping strategies at an individual and a dyadic level decrease under stress, leading to an augmented risk of physical and psychological aggression [9–11]. However, this vulnerable period is not limited to the time between conception and birth. Researchers have clearly pointed out that IPV with pregnancy-associated risk factors, encompass the time of 1 year before conception until 1 year after childbirth [4, 10, 12–14].

In terms of prevalence rate, a extensive range of pregnancy associated IPVv prevalence rates, varying from 3 to 30%, have been reported. Victimisation prevalence rates in African and Latin American countries are mainly situated at the high end of the continuum and the European and Asian rates at the lower end. Though estimates are highly variable due to methodological challenges, the majority of studies find rates within the range of 3.9% to 8.7%, with most studies merely including physical and/or sexual partner violence victimisation as psychological/emotional violence remains difficult to demarcate and measure [4, 8, 10, 12–20]. In Belgium, we recently reported [20] that 15.8% (95% CI 14.2 – 17.7) of the women experienced IPVv (incl. Psychological abuse) before and/or during pregnancy.

In recent years, research across the western world and increasingly in low- and middle-income countries has generated growing evidence that experiencing violence (as victim as well as perpetrator) in the perinatal period is related to risk behaviour and detrimental effects on the physical and mental health of women, men and children [21–28]. A cohort study of women aged 18–44 years suggested that IPVv was responsible for 7.9% of the overall burden of disease, which was more prominent than other risk factors such as high blood pressure, tobacco, and obesity [29, 30]. IPVv is hence considered an important contributor to the global burden of disease for women of reproductive age. In fact, IPVv during pregnancy and postpartum is more common than several maternal health conditions (e.g. pre-eclampsia, placenta praevia) with comparable negative consequences, and yet still IPV remain under-discussed within perinatal care [3, 4, 31, 32]. Most researchers and caregivers agree that perinatal care is an ideal moment to address IPVv, for it is often the only time in the lives of many couples when there is regular contact with health care providers [3, 33]. There is a growing consensus that routine screening is a safe effective practice and an important first step in tackling IPVv [34–39]. Nevertheless, much remains unclear concerning how to address IPV in the perinatal care context and which interventions should ideally be adopted. Despite greater recognition of IPV as a major public health problem, much less effort has been made to develop interventions aimed at decreasing IPV or its consequences [35, 40]. A number of systematic reviews [36, 41–43] have concluded that there is insufficient evidence supporting specific interventions for women experiencing IPV, especially those provided in health care settings. In line with these studies, our recent research results similarly suggested that specifically during the perinatal period, strong evidence of effective interventions for IPV is lacking [44]. The limited available evidence indicates that providing psychosocial support, advocacy, and suitable referrals to social and legal resources can potentially help women reduce their risk of violence and its consequences, and improve birth outcomes [45–47]. McFarlane and colleagues found that in a non-pregnant US population, disclosure of abuse was associated with the same reduction in violence and increase in safety behaviours as an intensive nurse case management intervention. According to these authors, simple assessment of abuse and provision of referrals have the potential to stop and prevent recurrence of IPVv and associated trauma. Inspired by this finding, we decided to investigate the effects of identifying IPV and distributing a referral card on the evolution of IPV, psychosocial health, help-seeking and safety behaviour within a pregnant Belgian population.

## Methods

### Setting and study population

We conducted a multi-centre single-blind Randomized Controlled Trial (RCT) in Flanders, the Northern part of Belgium. CONSORT reporting guidelines for RCT’s were followed. The trial consisted of two phases: 1) a prevalence study involving the recruitment of participants for the intervention; and 2) the intervention study. The methodology in the current article is similar to the methodology that has been published in Van Parys et al. (2014; 2015) [48].

The Belgian perinatal health-care system is based on the medical model [49] and is considered to be very accessible, with women freely choosing their own care provider(s). Obstetricians-gynaecologists merely function as primary perinatal healthcare providers and the majority of the care is hospital-based. Systematic inquiry or screening for IPV is not part of routine perinatal care.

We recruited in 11 antenatal care clinics to obtain a balanced sample of the general obstetric population. The convenience sample of hospitals included a mix of rural and urban settings, included small and large hospitals that provide services to socio-economically and ethnically diverse populations and was geographically spread across Flanders.

Women seeking antenatal care from June 2010 to October 2012, were consecutively invited to participate in the study if they were pregnant, at least 18 years old and able to fill in a Dutch, French or English questionnaire (cf. Additional file 1). We did not impose limits on gestational age. The midwife or receptionist introduced the study as a study on difficult moments and feelings during pregnancy and briefly explained the procedure. Women that orally consented to participate were handed an informed consent form and a questionnaire, which were both filled in in a separate room (if available) without the presence of any accompanying person. If the woman was unable to fill in the informed consent form and questionnaire in private, she was then excluded from the study for safety reasons. On the first page of the questionnaire women received an invitation to participate in the intervention phase of the study. Those willing to participate wrote their contact details down and were informed that eligible respondents would be interviewed twice by telephone and received a gift voucher as compensation. The selection of eligible participants for randomization was based on IPVv disclosure and willingness to participate in the intervention study. As a consequence, the IPVv prevalence rate at follow-up should have been 100%. However, 5 women were just below the victimisation threshold handled (see below) but slipped through the net of randomization, however thus were excluded from the final analysis.

The study was approved by the ‘Ethics Committee of Ghent University’ which acted as the central review board (Belgian registration number 67020108164) and by the local ethical committees of all 11 participating hospitals (Ethisch Comité Middelheim Ziekenhuis Netwerk Antwerpen, Ethisch Comité Universitair Ziekenhuis Antwerpen, Ethisch Comité Onze Lieve Vrouw Ziekenhuis Aalst, Ethisch Comité Gasthuis Zusters Ziekenhuis St Augustinus Antwerpen, Ethisch Comité Algemeen Ziekenhuis Sint Jan Brugge, Ethisch Comité Algemeen Ziekenhuis Jan Palfijn Gent, Ethisch Comité Onze Lieve Vrouw van Lourdes Ziekenhuis Waregem, Ethisch Comité Universitair Ziekenhuis Gent, Ethisch Comité Algemeen Ziekenhuis Groeninge Kortrijk, Ethisch Comité Virga Jesse Ziekenhuis Hasselt, Ethisch Comité Ziekenhuis Oost-Limburg Genk). The trial was registered with the U.S. National Institutes of Health ClinicalTrials.gov registry on July 6, 2010 under identifier NCT01158690) (https://clinicaltrials.gov/ct2/show/NCT01158690?term=van+parys&rank=1).

### Allocation concealment / randomization

As soon as the baseline assessment was filled in, the contact details and the related data of eligible respondents were systematically entered into an Access database. Case numbers were randomly assigned to the IG (intervention group) and CG (control group) by a computer generated list. The identification key was created and safely stored by a researcher not directly involved in the study.

At the postpartum consultation (+/− 6 weeks after delivery), the participants were handed a numbered opaque envelope. The lay-out and format of the envelopes of both groups were identical, so neither the health care providers nor the researchers could see or feel the difference. Since the envelope contained a referral card for the IG and a “thank you” card for the CG, it was not possible to blind the participants as a consequence of the design of this RCT. Nevertheless, we made a number of deliberate efforts to minimise the possibility of contamination between the two groups. First, the midwives/receptionists involved in the recruitment were not involved in the design of the study and had no knowledge of the hypotheses. Information about the study given to the clinical staff and receptionists was kept to a strict minimum. Second, women were allowed a separate available room where they filled in the questionnaire and waiting time at the clinic was minimised so that the intervention and control group women had little time or opportunity to meet each other. Moreover, the receptionists/midwives/doctors delivered the anonymous intervention or control envelopes to the women individually at postpartum check-up. Finally, the women’s allocation was not recorded anywhere, except in the secured identification key.

**Fig. 1:**
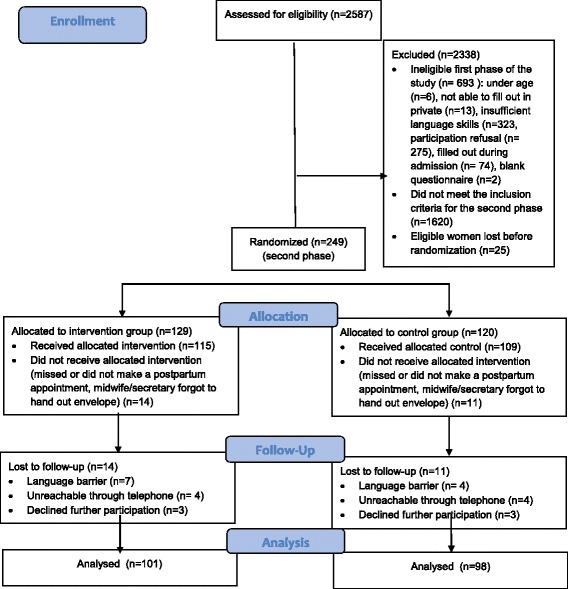
CONSORT flow diagram recruitment

In total 2,587pregnant women were invited to participate and 2338 were excluded of which 693 were ineligible for the first phase of the study, while 1620 did not meet the inclusion criteria for the second phase and 25 were lost before randomization. A total of 249 women were randomized, 129 allocated to the IG and 120 to the CG. At this stage, an additional 25 women were lost, and 10.9% in the IG and in the CG this was 9.2% did not receive the envelope due to the lack of a postpartum consultation or the oblivion of the midwife/receptionist. At the first follow-up interview (cf. Additional file 2) (10-12 months after receipt of the envelope), 12.2% was lost to follow up in the IG and 10.1% was lost in the CG, resulting in a final sample size of 101 in the IG and 98 in the CG. More details are presented in Fig. 1.

### Sample size

Since IPVv was the only main outcome measure with hard data available, the sample size was powered to test a reduction in the prevalence of IPVv. Calculations were based on the most recent prevalence estimate of IPVv in a Belgian pregnant population, which reported 3.4% physical and/or sexual partner violence in the year before and/or during pregnancy [19]. Since we measured IPVv several times [50] and also included psychological abuse, we expected to detect a prevalence that exceeded the most recent prevalence rate with 5%, equalling an total estimate of 8.4%.

Based on other RCTs with a similar study design, we considered an IPVv decrease of 30% relative to the 100% baseline prevalence in the IG clinically relevant, and we also hypothesised a 10% spontaneous or unexplained decrease of IPVv in the CG [33, 51, 52]. Assuming 30% loss to follow-up of and an alpha significance level of 0.05, at least 89 participants had to be included in each group (total *N* = 178) in order to detect a difference of 0.2 with 80% power. This means that a total sample of 2119 women was needed to retain the required number of women in both groups.

### Intervention

In brief, our study-intervention consisted of three parts: a questionnaire, a referral/thank-you card, and two interviews. Eligible women were handed an envelope by the midwife or receptionist at their 6-week postpartum consultation. The envelope of the IG contained: an information letter, a bank card-sized referral card containing the contact details of services providing assistance for IPV on one side and tips to increase safety behaviour on the other side, and a gift voucher. The resources and safety tips were selected in close collaboration with other researchers and expert care workers active in the field of IPV. The envelope of the CG contained: an information letter, a bank card-sized thank-you card, and a gift voucher.

**Fig. 2:**
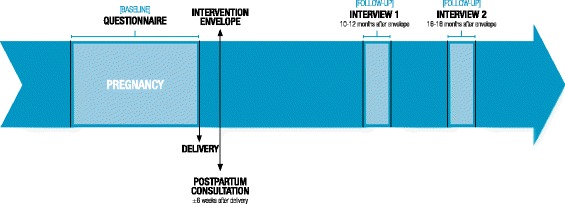
Time line study process

The participants were interviewed 10 to 12 months and 16 to 18 months after receipt of the envelope. The optimum period for the outcome measurement for this type of intervention has not been established. While some interventions may produce immediate positive effects, other effects may not be evident for some time. Therefore, we decided to time the first outcome measurement in a short term (within 12 months) and the second measurement in a medium term (from 12 to 24 months), as defined by Ramsey et al. [53]. Due to the large amount of data, this paper will be limited to reporting results of the first follow-up assessment at 10 to 12 months. Figure 2 provides an overview of the study process.

The information about IPV and resources for IPV provided to the health care professionals and receptionists in the participating hospitals was kept to a strict minimum, since the study aimed to measure the effect of the intervention in an unbiased manner with least intention to encourage help from the professionals in this stage. Furthermore, to our knowledge, only one in 11 participating hospitals displayed a sensitization poster and some folders concerning IPV. This led us to the assumption that the impact of parallel interventions on our respondents was minimal.

### Measures

The primary outcome measure of this intervention study was IPVv, and the secondary were psychosocial health, (in)formal help-seeking behaviour and safety behaviour. The full versions of all measures used are available in the supporting information. For the analysis of this paper, we used data from the baseline assessment and from the (first) follow-up assessment.

#### Baseline assessment

The baseline assessment essentially involved the assessment of physical, psychological, sexual IPVv and psychosocial health. In particular, **physical and sexual violence** was measured using an adapted version of the Abuse Assessment Screen [54]. For p**sychological abuse** we used an adapted version of the WHO-questionnaire [17]. Contrary to the situation for physical and sexual violence, currently there is a lack of consensus on standard measures and thresholds for psychological (partner) abuse/violence [1]. In an effort to address this problem we constructed a scale consisting of 7 questions with the answer options ranging from 0 to 4; total score obtained ranged between 0 and 28. Based on the limited available literature [1, 17, 47, 55–60] and after considerable debate and extensive consultations with several experts in the field, we did not consider a one-time minor psychological act as IPV and decided to use a cut-off value of 4/28 for psychological abuse. Hence, a score of 3 or lower was not considered psychological abuse to the purpose of this study.

**Psychosocial health** was measured through the Abbreviated Psychosocial Scale [61], which is well validated and is recently identified as the best currently available instrument for measuring multiple psychopathological symptoms [62]. The 28-item abbreviated psychosocial health scale consists of 6 subscales: negative affect (depression), positive affect (anxiety), positive self-esteem, low mastery, worry (anxiety) and stress. If data for one item was missing, the total score is considered as a missing value. A minimum score of 28 indicates ‘poor’ psychosocial health and a maximum score of 140 signifies ‘good’ psychosocial health. Unfortunately, to our knowledge no clear clinical cut-off value is currently available and therefore we used the scale as a continuous variable where possible. We have previously reported more details on the assessment of the violence [20] and on psychosocial health measures [48].

#### Follow-up assessment

The variables that were measured in the follow-up assessment are: socio-demographics (age and mother tongue), IPV (victimisation & perpetration), psychosocial health, help-seeking behaviour, readiness to change, safety behaviour, and helpfulness of intervention (questionnaire/referral card/interview). **IPV** was measured through the short form of the revised Conflict Tactics Scale (CTS2S) [63]. Although the CTS was intended as a self-report instrument, it can also be administered as a telephone interview [64]. The revised short form of the scale consists of 10 questions formulated in the form of paired questions (what the participant did = perpetration and what the partner did = victimisation). The questions address the issues concerning negotiation, physical assault, psychological aggression, injury from assault and sexual coercion. The response categories reflect the number of times that a certain aggressive behaviour took place over the last 6 months. If data for one item was missing, the total score was considered a missing value. There are several ways of analysing the CTS2S. We chose to use the score as a dichotomous variable for most analyses and used the severity levels (minor/severe) to test if the referral card would be more effective in women experiencing severe IPVv. Based on the authors’ scoring instructions, respondents who indicated a certain behaviour (except for negotiation) taking place at least once are considered to have experienced IPV (as a victim and/or as a perpetrator). This implies that a one-time minor act of psychological aggression, e.g. ‘your (ex)partner insulted you, or swore, shouted or screamed at you’, will yield a positive score. Although the CTS2S also measures perpetration behaviour of the women included in the study, the main analysis for this paper is based on victimisation. In comparison with the threshold for IPVv at baseline, we did not include a one-time minor act of psychological aggression in the follow-up measurement and set the threshold at 3 - 5 incidents (in the last 6 months). The combination of both violence measures, the AAS as a quick identification scale and the CTS as a more in-depth measure, is a widely used practise in many intervention studies [65].

**Psychosocial health** was assessed using the same scale, namely the Abbreviated Psychosocial Scale as in the baseline survey, yet with adaptations made for a telephone interview.

Measurement of **formal and informal help-seeking behaviour** was based on an adapted version of ‘Community agencies use questionnaire’ developed by McFarlane et al. and Fanslow et al. [66]. Both variables were dichotomized, with contacting at least one agency or person being classified as a positive score for help-seeking behaviour. Additionally, we explored causes or reasons for seeking or not seeking help. The answers to these open questions were grouped in large categories and quantified to gain an overview of the most frequently cited reason to seek formal help.

**Readiness to change** is introduced as a mediating variable for help-seeking behaviour, since it is known that seeking help is influenced by the phase in which people are located [66–68]. The answer that indicates not considering making any changes to the situation in the next 6 months was coded as the precontemplation phase. In contrast, considering making changes in that space of time was coded as the contemplation phase, while thinking about making changes in the next 30 days was coded as the preparation phase.

**Safety behaviour** was based on an adapted version of the ‘Safety promoting behaviour checklist’ [33]. A positive answer to at least one safety behaviour question, obtained a positive dichotomised score.

The degree of **helpfulness of intervention** (questionnaire/referral care/interview) was dichotomised into ‘somewhat or very helpful’ and ‘not helpful or made things worse’.

The interview was available in Dutch, French and English and was based on a translation and back- translation of the original instruments.

### Statistical analysis/data-analysis

The data obtained through the first interview were first recorded on paper in a structured form by the interviewer and then entered into an SPSS (Statistical Package Social Sciences) database by another researcher who also performed quality control and data cleaning.

A descriptive analysis was performed for both study-arms regarding socio-demographic data, IPV, psychosocial health, formal and informal help-seeking behaviour, readiness to change, safety behaviour and perceived helpfulness of the intervention. Baseline socio-demographic characteristics and psychosocial health were compared between both study-arms using an independent two samples T-test for the continuous variables and a Fisher’s exact test for the categorical variables.

The evolution of IPV from baseline to follow-up interview 1 was investigated using a McNemar test. The difference in IPV prevalence at follow-up between IG & CG was assessed based on a binary logistic regression model, thus adjusting for significantly different baseline characteristics between both study-arms. The evolution of psychosocial health from baseline to follow-up interview 1 was measured through a paired T-test, for the group as a whole and for both study-arms. A general linear model (unianova) was employed to explore the difference between the IG & CG for psychosocial health at follow-up, adjusting for psychosocial health at baseline. We also used Fisher’s exact tests and corresponding 95% Wilson’s score statistic CI for difference of two independent proportions, and multiple logistic regression adjusting for psychosocial health at baseline to assess the differences between the IG and CG for formal and informal help-seeking behaviour, safety behaviour and perceived helpfulness of the intervention.

The main data analysis was based on a complete case analysis, followed by a sensitivity analysis which examines the robustness of the results regarding to missing data, especially since it is known that women lost in IPV-studies are more likely to be abused [69, 70]. Different scenarios were studied with IPV as the main outcome variable. For instance, the ‘best scenario’ refers to the situation in which all the women lost in the study happened not to report IPVv, the ‘worst scenario’ was related to the possibility that all the women lost in the study did report IPVv and we also explored the ‘Last Observation Carried Forward’ (LOCF).

In the sensitivity analysis, missing baseline IPV data were replaced by a positive IPVv score, since IPVv was an inclusion criteria for the intervention study.

All statistical analyses were performed using the IBM SPSS statistics software (version 23).

## Results

### Socio-demographic data

Table 1 provides an overview of the baseline and follow-up socio-demographic characteristics of the respondents at an average of 10 months (Standard Deviation (SD) 1 month) after receipt of the envelopes.

**Table 1:** Socio-demographic characteristics of the sample (*n* = 223)^a^

Characteristics baseline assessment		IG (*n* = 115)	CG (*n* = 108)	*P* value
Age in years (SD)		27.87 (4.98)	27.67(5.39)	0.771
		IG (*n* = 112)	CG (*n* = 103)	
Gestational age in weeks (SD)		23.63 (8.35)	24.57 (8.28)	0.405
Civil/marital status		**n = 114** **% (n)**	**n = 106** **% (n)**	0.677
	Married/cohabiting	87.7 (100)	89.6 (95)	
	Single/Divorced	12.3 (14)	10.4 (11)	
Education		**n = 114** **% (n)**	**n = 107** **% (n)**	0.929
	None/primary education	12.3 (14)	13.1 (14)	
	Secondary education	44.7 (51)	42.1 (45)	
	Higher education	43 (49)	44.9 (48)	
Language questionnaire		**n = 114** **% (n)**	**n = 109** **% (n)**	1.000
	Dutch	93 (106)	92.7 (101)	
	French	1.8 (2)	1.8 (2)	
	English	5.3 (6)	5.5 (6)	
Characteristics 10-12 months assessment		**IG** (**n = 101**)	**CG** (**n = 96**)	
Age in years (SD)		29.7 (4.78)	29.15 (5.21)	0. 436
Mother tongue		*n* = 101 % (n)	*n* = 96 % (n)	0.181
	Dutch	80.2 (81)	87.5 (84)	
	Not-Dutch	19.8 (20)	12.5 (12)	

^a^baseline data for one woman was lost

After unblinding the raw data, we compared the key baseline characteristics of the IG and CG, to check if the randomization was successful. No significant differences were found between the socio-demographic characteristics of both groups (cf. Table 1). However, psychosocial health differed significantly at baseline (*P =* 0.044), with the mean psychosocial health in the CG being 98.85/140 (SD 14.92) and in the IG 103.36/140 (SD 15.89). Accordingly, the multivariate analyses for main outcome variables were adjusted for baseline psychosocial health.

### IPV

At follow-up (*n* = 189), 66.7% (*n* = 126) of the participants reported IPVv and 63% (*n* = 119) reported IPVp. Accordingly, the prevalence of IPVv in the entire cohort decreased to a significant extent [31.4% (95% CI 24.5; 38.7), *P* < 0.001] at the postpartum assessment, though this trend did not differ between both study arms [IG: 32.6% (95%CI 22.5; 43.1) and CG: 30.1% (95% CI 20.8%; 40.4), *P* = 0.644]. Adjustment for psychosocial health did not alter the results [OR 1.13 (95% CI 0.58; 2.2), *P* = 0.727].

Table 2 presents an overview of the IC/CG comparison for the main outcome variables.

**Table 2:** Overview results main outcome variables

Outcome (dichotomous)	IG % (*n*)	CG % (*n*)	Difference (95% CI) IG-CG	*P*-value	Unadjusted OR IG vs CG (95% CI)	*P*-value	Adjusted^a^ OR IG vs CG	IG (*n*)	CG (*n*)	*P*-value
IPV baseline	98.2% (112/114)	99.1% (108/109)	−0.8% (−5.4;3.4)	1.000	0.51 (0.05;5.80)	0.594	0.48 (0.04; 5.53)	99	92	0.559
IPV follow-up	64.6% (62/96)	68.8% (64/93)	−4.2% (−17.5;9.2)	0.644	0.83 (0.45;1.52)	0.537	1.13 (0.58; 2.20)	85	80	0.727
Formal help seeking behaviour	19.4% (19/98)	28.4% (27/95)	−9.0% (−21.1; 3.1)	0.177	0.61 (0.31; 1.18)	0.143	0.76 (0.37; 1.58)	87	81	0.466
Informal help-seeking behaviour	65.3% (64/98)	75.8% (72/95)	−10.5 (−23.1; 2.4)	0.118	0.60 (0.32;1.13)	0.112	0.71 (0.36; 1.40)	87	81	0.326
Safety behaviour	24.5% (24/98)	38.3% (36/94)	−13.8% (−26.6; −0.7)	*0.044*	0.52 (0.28;0.97)	0.040	0.76 (0.38; 1.51)	87	80	0.431
Helpfulness questionnaire	36.1% (35/97)	38.9% (35/90)	−2.8% (−16.6; 11.0)	0.763	0.89 (0.50;1.60)	0.692	0.83 (0.43; 1.58)	86	76	0.568
Helpfulness referral card	28.9% (28/97)	17.6% (16/91)	11.3% (−0.9; 23.2)	0.085	1.90 (0.95;3.82)	0.070	1.98 (0.94; 4.18)	86	77	0.072
Helpfulness interview	36.1% (35/97)	34.1% (30/88)	2.0% (−11.8; 15.6)	0.878	1.09 (0.60;2.00)	0.777	1.21 (0.62; 2.34)	86	75	0.578
Outcome (continuous)	Mean score IG	Mean score CG	Difference score (95% CI) IG-CG	P-value			Adjusted^a^ mean difference score (95% CI) IG-CG			P-value
Psychosocial health baseline (score on 140)	103.36	98.85	4.52 (0.12; 8.91)	*0.044*	/	/	/	/	/	/
Psychosocial health follow up	105.81	105.26	0.55 (−5.00; 6.07)	0.845	/	/	3.29 (−1.85;8.42)	84	79	0.208

^a^Adjusted for psychosocial health at baseline

Sensitivity analysis provided no arguments that missing data affected this comparison, 73.6% of the women in the IG and 75.8% in the CG reported IPVv (*P* = 0.771) for the LOCF as well as for the worst scenario which assumes all women with missing data did report IPVv. In the best scenario that assumes all women with missing data did not report IPVv, 48.1% reported IPVv in the IG and 53.3% (*P* = 0.447) in the CG.

### Psychosocial health

Mean scores (*n* = 163) for psychosocial health overall increased significantly by 5.4 points (95% CI 2.6; 8.2, *P* < 0.001) over the study period (baseline: 101.44; SD 16.07 and follow up: 106.83; SD 18.72), though this evolution was only significant in the CG [IG: 2.9 (95% CI -0.7; 6.5), *P* = 0.113 and CG: 8.1 (95% CI 3.8; 12.3), *P* < 0.001]. After adjustment for psychosocial health at baseline, a significant improvement in mean psychosocial health score was retained, though no longer between both study arms (*P* = 0.208).

### Help-seeking behaviour

#### Formal

The majority (76.2%, *n* = 147/193) of the women in our sample did not contact any service providing assistance in dealing with problems with their partners in the last 6 months, and 23.8% (*n* = 46) contacted one or more services. The maximum number of services contacted by women was 5. Table 3 provides an overview of the types of formal services that were contacted. The descriptive data show that first and foremost the women contacted legal services and the police, then psychological and social services.

**Table 3:** Overview formal services contacted

Contacted services	% (*n*)
Legal services (legal aid, lawyer, court, …)	10.9% (21)
Police	8.3% (16)
Psychologist^a^	7.3% (14)
Centre for General Welfare (Centrum Algemeen Welzijn)	4.7% (9)
Mental Health Centre (Centrum Geestelijke Gezondheid)	3.6% (7)
Hospital Social Services	3.6% (7)
Family physician^a^	3.6% (7)
Psychiatrist ^a^	1.6% (3)
Women’s shelter or safe house (Vluchthuis)	1.6% (3)
Social Service Department municipality (Openbaar Centrum Maatschappelijk Welzijn)^a^	1.6% (3)
Youth Welfare Service (Comité Bijzondere Jeugdzorg)^a^	1.0% (2)
Victim Support Service (Slachtofferhulp)^a^	0.5% (1)
Service for Assisted Living (Begeleid Wonen)^a^	0.5% (1)
Marriage of convenience Cell (Cel Schijnhuwelijken)^a^	0.5% (1)
Preventive Family Welfare Agency (Kind & Gezin)^a^	0.5% (1)
Moral Support Service (Huis van de Mens)^a^	0.5% (1)
Gynaecologist^a^	0.5% (1)
Telephone support hotline (Tele-onthaal)	0.5% (1)
Self-help group	0% (0)

^a^mentioned by the respondents under the option “other”

In the IG, 19.4% (*n* = 27/95) of the women sought formal help and 28.4% (*n* = 27/95) did so in the CG (*P* = 0.177). Adjusted for psychosocial health at baseline, the difference in formal help-seeking behaviour between the IG and CG remained insignificant (*P =* 0.466). More details are available in Table 2. The most frequently cited reason (88.6%) for not seeking help was that it was not perceived it as ‘necessary’.

Women reporting IPVv did seek significantly more formal help (31.0%, *n* = 39/126), compared to those not reporting IPVv (9.5%, *n* = 6/63) *(P* = 0.001). Similarly, women reporting IPVp sought considerably more formal help (29.4%, *n* = 35/119), compared to those not reporting IPVp (14.3%, *n* = 10/70) *(P* = 0.021).

#### Informal

Our findings indicate that 70.5% (*n* = 136/193) of the women talked to someone about the IPV, outside of the formal services assessed. The large majority of women spoke to family (49.7%, *n* = 96/193) and friends (47.7%, *n* = 92/193).

In the IG, 65.3% (*n* = 64/98) of the women sought informal help and 75.8% (*n* = 72/95) did so in the CG (*P* = 0.118). After adjusting for psychosocial health at baseline, informal help-seeking behaviour was not different in the IG compared to the CG (*P* = 0.326). More details can be found in Table 2.

When women reported IPVv, they sought substantially more informal help (78.6%, *n* = 99/126) compared to those not reporting IPVv (54.0%, *n* = 34/63) (*P* = 0.001). If women reported IPVp, they also sought significantly more informal help (79.0%, *n* = 94/119) compared to those not reporting IPVp (55.7%, *n* = 39/70) (*P* = 0.001).

In comparing the women’s formal with informal help-seeking behaviour, they sought considerably more informal one (*P* < 0.001).

#### Readiness to change

Over half of our respondents (57.1%, *n* = 109/191) did not consider making changes to their relationship in the next 6 months (precontemplation phase), while 15.2% (*n* = 29/191) of the women considered making changes (contemplation phase) and 27.7% (*n* = 53/191) of the women thought about making changes in the following months (preparation phase). There was no statistical difference in readiness to change between the IG and the CG (*P* = 0.159).

Formal and informal help-seeking behaviour was statistically significantly correlated to being in more advanced phase of the readiness to change process (*P* < 0.001 and *P* = 0.010). After Bonferroni correction a significantly higher proportion of formal and informal help-seeking behaviour was found in the preparation phase compared to the precontemplation phase (*p* < 0.001 and *p* = 0.007).

### Safety behaviour

The results for safety behaviour indicate that 31.3% (*n* = 60/192) of the women took one or more safety measures. The majority of the women made sure to have a small amount of money with them in case of emergency (25.4%, *n* = 49/193), 10.9% (*n* = 21/193) stored an emergency bag (clothes, spare keys etc.) in a safe location, 8.3% (*n* = 16/192) agreed on a code with someone who will then call the police, and 2.1% (*n* = 4/192) removed objects that could be used as a weapon.

In the IG, 24.5% (*n* = 24/98) of the women took one or more safety measures and 38.3% (*n* = 36/94) did so in the CG, with significantly more safety behaviour in the CG (*P* = 0.044). Adjusted for psychosocial health at baseline, the difference between the IG and the CG was no longer significant (*P* = 0.431). More details are available in Table 2.

We found significantly more safety behaviour when women reported IPVv (37.3%, *n* = 47/126) compared to those not reporting IPVv (17.7%, *n* = 11/62) (*P* = 0.007).

### Perceived helpfulness of the intervention

More than a third of the women considered the questionnaire (37.4%, *n* = 70/187) and/or the interview (35.1%, *n* = 65/185) to be reasonably to very helpful. Only one woman indicated that filling in the questionnaire made things worse. The referral card was rated as somewhat to very helpful for 23.4% (*n* = 44/188) of the women.

The questionnaire was helpful for 36.1% (*n* = 35/97) of the IG and for 38.9% (*n* = 35/90) in the CG (*P* = 0.763). As far as the usefulness of the referral card is concerned, the proportion was 28.9% (*n* = 28/97) in the IG and 17.6% (*n* = 16/91) in the CG (*P* = 0.085). In regard to the interview 36.1% (*n* = 35/97) in the IG and 34.1% (*n* = 30/88) in the CG (*P* = 0.878) rated it helpful. Adjusted for psychosocial health at baseline, the differences in helpfulness between IG and CG remained insignificant (*P* = 0.568, *P* = 0.072, *P* = 0.578). Based on these results, the helpfulness of the referral card appeared to be greater in the IC, although it borderline missed statistical significance. More details are available in Table 2.

In the whole sample, the perceived helpfulness of the questionnaire (37.4%, *n* = 70/187) and the interview (35.1%, *n* = 65/185) were both significantly larger compared to that of the referral card (23.5%, *n* = 44/187) (*P* < 0.001). We did not find a significant difference in perceived helpfulness between the questionnaire (37%, *n* = 68/184) and the interview (35.3%, *n* = 65/184) (*P* = 0.368).

Lastly, we hypothesised that the referral card would be more effective in women experiencing severe IPVv due to a more urgent need for help; our dataset, however, did not provide any evidence showing that severity of IPVv had a significant effect on the intervention (*P* = 1.000).

## Discussion

### IPVv

First, we found a statistically significant decrease of IPVv of 31.4% over the study period, although we are unable to attribute this decrease to the referral card. Compared to other studies with a similar design where most authors consider a decrease of 20% clinically relevant [45, 53, 59, 71, 72] we consider our decrease of IPVv over the course of the study pertinent. A significant reduction in IPVv prevalence rates over time, regardless of the type of treatment, is consistent with findings from other intervention studies conducted in a variety of social and health settings. Another important finding is that there appear insignificant differences between intervention and control groups, which is in line with that of Cripe et al. [22], Zlotnick et al. [72], Curry et al. [73], Humphreys et al. [74]. However, some RCTs, which evaluate home visit programs [27, 69, 75, 76] and typically address several issues (e.g. psychosocial health, parenting skills, substance abuse) simultaneously, showed promising results and reported a significant decrease in physical, sexual and/or psychological partner violence (odds ratios from 0.38 to 0.92) in their intervention groups. The Dutch equivalent of the Olds et al. home visit program [76] reported significantly less IPV victimisation and perpetration in the IG until 24 months after birth in a sample of high-risk young pregnant women. Evidence from another two studies examining different types of supportive counselling [45, 59] also supported a statistically significant effect of their intervention. More specifically, in the 30-min one-to-one session from Tiwari et al. [59] significantly less emotional and minor physical (except for sexual IPV) violence was reported in the IG. Kiely et al. [45] concluded that their comprehensive cognitive behavioural intervention reduced recurrent episodes of IPV (again except for sexual IPV) significantly.

It has been previously hypothesised that the decrease in IPV prevalence rates, regardless of the fact if there is a difference between the intervention and the control groups, may be attributed to a simple regression toward the mean or natural history of IPV, which may wax and wane. Since there is no clear evidence-based indication of the optimum period of outcome measurement for this type of intervention, it might be possible that we missed the immediate positive effects or other effects that may not have been evident for some time. At the time of measurement, the respondents simply might not acknowledge the violence as such, or be ready to make changes, seek or accept help. Some counselling interventions (e.g. distributing a referral card, undertaking safety measures, developing safety plans, or seeking help) might come too early/late and/or are not adapted to specific needs at given time and therefore prove ineffective [66, 77, 78]. Furthermore, the choice of decrease of IPV as one of the main outcome variables to measure the impact of the intervention may not have been the most appropriate outcome measure. An increasing number of studies have shown that IPV generally involves a complex process, given the numerous steps and intervening factors between identification and IPV reduction, many of which are beyond the control of the health care system or providers. Therefore, interventions should not necessarily be expected to reduce IPV. Other measures of internal change, such as psychosocial health and quality of life, have been suggested as potentially more informative for evaluating the impact of an intervention for IPV [39]. However, the significant improvement of psychosocial health identified in our study cannot be explained by the referral card either.

Another hypothesis for the insignificant difference between the IG and CG is that the design of the intervention might not have been adapted to the type of IPV found in our study. Based on Johnson’s [79] typology we can distinguish 2 types of violence: ‘mutual violence’ and ‘intimate terrorism’. In brief, the interpersonal dynamic in mutual violence is one of conflict that escalates to minor low-frequency forms of violence where either or both partners can be violent. Fear is not a characteristic of mutual violence and most couples deal with it themselves. In intimate terrorism, the (usually male) perpetrator uses violence as a tactic in a general pattern of power and control over his partner who does not resort to violence. This type of violence is likely to escalate over time, less likely to be mutual and more likely to result in injuries to women and draw attention from neighbours, police and health caregivers. Our study design did not differentiate these two types of violence, however, there is indication that we might have mainly included low severity ‘mutual violence’. First, our results show that the number of women reporting victimisation and perpetration of IPV, respectively, is fairly close, with 66.7% (*n* = 126) compared with 63% (*n* = 119). This is similar to the findings of other authors (e.g. Bair-Merritt et al. [27]). Second, we have reported earlier that only a very small proportion (1.2%; *n* = 22) of the women indicated being afraid of the perpetrator. Third, most women were dealing with the IPV themselves, as reflected in the findings that less than one fifth (22.6%; *n* = 40) contacted at least one formal service and the most frequently cited reason for not seeking any formal help was that it was perceived as ‘unnecessary’. However, besides referring to light forms of IPV, this notion of ‘not necessary’ could also refer to the denial or minimization associated with the precontemplation phase. Based on the structure of our intervention, which centres around IPV assessment and distribution of a referral card in order to reduce IPV and improve psychosocial health/help seeking and safety behaviour, it is plausible that this type of intervention is rather directed towards addressing ‘intimate terrorism’ instead of ‘mutual violence’. Moreover, the intervention did not directly involve the partner or concretely addressed female violent/abusive behaviour, which most probably are factors preventing the development of less abusive communication.

### Help-seeking behaviour

About a fourth of the women contacted one or more formal services. They contacted first and foremost legal services and the police, then psychological and social services. In contrast, 70.5% of the women opted for informal help and most of them talked to family and friends about the problems with their (ex)partner. Women reporting IPV victimisation and perpetration showed significantly increased formal and informal help-seeking behaviour.

The low use or the underutilisation of formal reources providing IPV-related assistance is in concordance with the findings of several other authors [37, 80, 81], although both we and Ansara & Hindin [82] have identified the police and health professionals as the commonly used formal resources. In a similar vein, literature has shown that informal sources of help and social support, including family, friends and coworkers, are the primary source women call upon to in dealing with IPV [83]. Several population-based studies have shown that 58% to 80% of abused women opt to share information about the abuse and seek support at least once with any informal resource [84].

Based on the stage model of help-seeking behaviour of Liang et al. [81], one could argue that people progress from making initially more private attempts to seeking informal support to deal with abuse, and as violence worsens, to pursuing more formal/public help [85]. This theory aligns with our assumption that we probably mainly measured low-level mutal violence with regard to which IPV is defined as temporary, survivable or reasonable and for which private attempts and informal help are used as main resources. Furthermore, Fanslow and Robinson [66] found that 63.4% of the abused women in their study did not seek help from formal services due to their perception of the violence to be ‘normal or not serious’. Similar to our findings that seeking formal help was perceived as ‘unnecessary’, this perception of ‘normality’ has resulted in women enduring violence without any (formal) help. Couples typically do not perceive low-level IPV as problematic in their relationship however, research has shown that they are at high risk for future relationship dissatisfaction and instability [63, 86]. Additionaly, we found that more than half of our participants were in the precontemplation phase according to the model on readiness to change [87], which implies that they were minimising or denying the IPV.

Several authors [53, 78, 88] have argued that women recruited in health care settings may differ from those recruited elsewhere, since they may not yet be at the stage of identifying their relationship as abusive or ready to accept help. Relationships between intimate partners involve a wide range of activities, ranging from eating, sleeping, co-parenting, playing, working, making major and small decisions, to sexual activity. The fluid and intimate nature of these interactions may make subtle violations and abuses difficult to detect and hard to understand or define. Moreover, because the actual nature, severity, and presence of violence in an intimate relationship may be constantly shifting, alternating between violence and loving contrition, acknowledging the relationship as abusive may be difficult and confusing [81]. If one does not identify the abuse/violence as such, one is unlikely to utilize resources. Knowledge, attitudes, and beliefs about abuse develop within sociocultural contexts and influence how women define and respond to experiences [84]. More specifically, the childbearing cycle strengthens the bonds between partners and their commitment to the family. For some women the pregnancy and safeguarding the child can be a catalyst to leave the relationship behind, whereas for other women pregnancy may weaken the ability to deal or cope with the IPV and stimulate them to find ways to reduce the violence or modify their own response to violence (e.g. refraining from fight back) [89, 90].

There are a range of other factors, e.g. the type, extent and severity of IPV that have been associated with help-seeking behaviour. Some authors argue that women experiencing more severe violence (involving the use of deadly objects or the fear for one’s life) seek more help [91], while others, as demonstrated in our results, do not reveal any sign of the connection between the impact of severity and their help-seeking behaviour [92]. Socio-demographics (including age, education, socioeconomic, and marital status) and psychosocial health have also been shown to influence help-seeking behaviour [91, 93–95]. Psychosocial dysfunction associated with IPV may negatively influence a woman’s help/health seeking behaviour [94]. In contrast, psychologically healthier individuals could be more likely or better equipped to reach out for help [93]. Hence, the low psychosocial health scores at baseline might have had an impact on the effect of the intervention.

### Perceived helpfulness of the intervention

Next, 37.4% of the women judged the questionnaire and 35.1% found the interview as moderately to highly helpful. The referral card was regarded by 23.4% as moderately to highly helpful. Although we were not able to detect significant differences between the intervention (*n* = 28) and the control groups (*n* = 16), the helpfulness of referral card seemed to be more prominent in the IG and approximated statistical significance (*p* = 0.085). Although in recent years, more and more evidence is emerging that low intensive interventions such as handing a referral card are not likely to have a large and lasting impact on women’s experience of IPV [96], the evidence based is still quite limited .

In contrast to McFarlane’s [33] suggestion, we are unable to conclude that the simple assessment of IPV, in combination with offering referrals, has the potential to interrupt and prevent recurrence of IPV and associated trauma. We found that the identification of IPV, together with the distribution of a referral card (compared to a thank-you card), did not result in a statistically significant difference of the measured outcomes in both arms.

We hypothesise that this finding is closely related to the very different organisation of the health care and social services systems in Belgium and the USA, as the organizational structures and systems are strongly embedded in the countries’ own cultural contexts. In that sense, it may be that in a society with a higher tolerance for violence (e.g. Belgium), the victims tend to regard their experiences as less offensive or abusive. Women might not acknowledge certain behaviours as being transgressive and consequently, feel hesitant to seek help. Conversely, the USA have a long tradition of condemning violence and women might be more easily stimulated to find help based on a referral card.

The women in our study perceived being asked about IPV as more helpful than receiving a referral card. Similarly, Chang [97] has shown that screening for IPV during pregnancy can help raise awareness and women’s interactions with health care providers may help change women’s perceptions. Health psychology has demonstrated that an effective means to change health related behaviour is to ask people questions about that behaviour (e.g. their intentions), as doing so influences the likelihood and rates of performing that behaviour [98]. Indeed, IPV assessment can have a therapeutic value on its own and that the process of measurement changes the very thing being measured [33, 99]. As described earlier, screening for IPV is not part of routine antenatal care in Belgium. A possible explanation for the perceived helpfulness of the questionnaire/interview is that being asked about IPV in a health research related context (also known as the Hawthorne-effect) might have had a greater impact than anticipated in both groups. Moreover, we cannot exclude the possibility that the study may have triggered some health professionals to pay more attention to IPV and might have increased their help-providing behaviour, although we assume that this behavioural change was limited and of short duration.

### Strengths and weaknesses

This study has a number of strengths and weaknesses. The recruitment took place in a balanced sample of 11 antenatal care clinics spread across Flanders (Belgium). Based on a sound sample size calculation, we were able to include a sufficiently large sample of women. Randomisation was successful for all variables except for psychosocial health, for which we adjusted in our analysis. The number of women lost to follow-up was limited and we found no statistical evidence that the missing data would have altered the main findings in this study. Yet, the prevalence rates reported are most probably an underestimation since it is know that women lost in IPV-studies are more likely to be experience IPVv. Furthermore, the exclusion of women who were not proficient in Dutch/French/English and were not able to fill out in private might have created a bias, although we assume that the impact is limited. Considerable efforts were made to ensure that women were able to fill in the questionnaire or be interviewed in private, but it is conceivable that a part of our respondents were under watch of their (abusive) partners. Another potential source of bias is that the women willing to participate in the study may have been more motivated or ready to seek help or install safety behaviours and take actions to reduce IPV, compared with women who did not consent to participate or were lost to follow-up. Furthermore, almost a fourth of the women indicated not having received or not recalling having received a referral card and might have produced a biased view on the impact of the intervention. Additionally, answering questions about the helpfulness of a questionnaire/interview in a telephone interview directly to the researcher self might have stimulated social desirability bias and skewed the answers towards increased helpfulness. Lastly, we did not control for measurement reactivity effects, which might have produced a more nuanced picture of the impact of the perceived helpfulness of identifying IPV.

## Conclusions

In this multicentre RCT we found a significant decline in the prevalence of IPVv and an increase of psychosocial health at follow-up, though we failed to document any additional effect of handing out a resource referral card in women disclosing IPVv during pregnancy. 70.5% of the women sought informal help and more than one fifth pursued formal help and. Women reporting IPV showed significantly increased formal and informal help-seeking behaviour. A third of the women took at least one safety measure, and safety measures were taken significantly more frequently when IPV was reported. The questionnaire as well as the interview in this study were perceived as moderately to highly helpful by more than a third of our sample and this degree of helpfulness was significantly greater than that of the referral card. We were unable to link any of the above findings directly to handing out the referral card. Although the helpfulness of the referral card appeared to be more substantial in the IC, it borderline missed statistical significance.

In other words, detection of even low severity mutual IPV can be a helpful tool in the fight against IPV, though the combination of identification with simply the distribution of a referral card is probably not the best means of achieving that goal.

Based on our results, we recommend that future intervention studies address simultaneously several risk factors such as for example psychosocial health, substance abuse, and social support. Intervening in a single risk factor, as with IPV in our case, might be unsuccessful because other risk factors may persevere as barriers to the desired change. We believe that comprehensive IPV interventions that address risk factors at the individual, interpersonal, societal, cultural and community levels concurrently have higher chances of success. Interventions that involve informal networks as a fundamental component might also be more effective. However, large-scale, high-quality research is essential for providing further evidence of the content of these interventions and for clarifying which interventions should be adopted in the perinatal care context. Furthermore, we recommend that future IPV interventions include information on the typologies of IPV (intimate terrorism and mutual violence) in their assessments. Doing so will allow researchers to accurately test and compare the effects of different types of IPV victimisation and perpetration among pregnant and postpartum women. Demarcating these groups and taking account of the stages of change, the help-seeking strategies and complex mutuality of IPV will offer great potential for designing a tailored intervention that is well adapted to the specific needs of couples experiencing IPV.

## Additional files

Additional file 1: Questionnaire. (PDF 1624 kb)

Additional file 2: Interview scheme. (PDF 466 kb)

## Abbreviations

CG: Control group
CTS2S: Revised conflict tactics scale
IG: Intervention group
IPV: Intimate partner violence
IPVp: Intimate partner violence perpetration
IPVv: Intimate partner violence victimisation
LOCF: Last observation carried forward
OR: Odds ratio
RCT: Randomized controlled trial
SD: Standard deviation
SPSS: Statistical package for social sciences
