# Supplementary material for: The impact of a referral card-based intervention on intimate partner violence, psychosocial health, help-seeking and safety behaviour during pregnancy and postpartum: a randomized controlled trial
Source: BMC Pregnancy Childbirth. 2017 Oct 6;17:346. doi: 10.1186/s12884-017-1519-x (PMC6389099; doi:10.1186/s12884-017-1519-x)
Supplement: Supplementary file 2 — Interview scheme. (PDF 466 kb) [file 12884_2017_1519_MOESM2_ESM.pdf]

ENGLISH Interview scheme 10-12 months

Good morning/afternoon/evening Ms …

My name is An-Sofie Van Parys. I am the midwife who is coordinating the study in which you took part in-------------------------------------(some time ago) in ---. During your pregnancy you filled in a questionnaire (about difficult times and feelings) and agreed to give an interview. That is why I am calling you now; the interview will last about 20 min.

- **Is this a good time for you?**
  - **If NO: when could I call you back?**
  - **If YES: ↓**
- **Are you alone right now?**
  - **If NO: When could I call you back, because it is very important for the study that you are alone [to avoid that you are distracted and I can ask some questions in private]?** *[Refuse emphatically if woman wants to continue in presence of other person(s): standard study protocol is that all women have to be alone, so answers are comparable]*
  - **If YES: should your (ex)partner or anyone else enter the room at any time, you can just hang up and I will call you back later [as it is important for the purpose of the study that you are alone]** ↓ *[I am the midwife of hospital -- and we are calling up everyone a few months after they have given birth to see how they are doing]. You seem to be very concerned over your wife and that is admirable, but would it be possible to talk to her over the phone?" It seems a bad time to call'*

With this interview, we want to better understand your experiences and feelings and look for better ways to <u>help pregnant women</u>.
I will be asking a number of <u>explicit questions</u>, which may be difficult to answer. Be aware that you are not obliged to respond and that you can stop at any time.
Everything you tell me is <u>strictly confidential</u>. This means that your name will not appear anywhere and everything will be processed entirely anonymously.
The idea is not for this to become a <u>therapeutic or counselling</u> session. If you were to need such a session after our talk, I would like to refer you to a local care worker. [CAW <u>078/150,300, www.caw.be, tele-onthaal 106/107]</u>

Do you have any <u>questions</u> before we start?

DEMOGRAPHICS

OK, then we'll start the interview with some general questions

D 1	What is your date of birth? (day / month / year)	— —/— —/— — — —	
D 2	Are you currently in a (partner) relationship?	0 No	1 Yes
D 2a	**If YES:** how long have you been together?	— — years<br>— — months	
D 2b	Have you had one or more (<u>other</u>) (ex)partners in the past 2 and a half years?	0 No	1 Yes
D 2c	How long were you together for?	1) — — months<br>— — years<br>2) — — months<br>— — years<br>3) — — months<br>— — years	
D 3	Who is currently part of your family? (children + adults)?	— —— —— —— —— —<br>— —— —— —— —— —<br>— —— —— —— —— —<br>— —— —— —— —— —<br>— —— —— —— —— —<br>Total number:	
D 3a	How old is your child / are your children?	— — years<br>— — years<br>— — years	
D 4	What is your mother tongue?	— —— —— —— —— —	

OBSTETRIC HISTORY

O 1	How many times have you been pregnant, i.e. including miscarriages and abortions?	—— times	
O 2	How many miscarriages have you had?	——	
O 3	How many abortions have you had?	——	
O 4	Are you pregnant at the moment?	0 No	1 Yes
O 5	When did you give birth most recently?	——/——/————	
O 6	Was this pregnancy planned?	0 No	1 Yes

PSYCHOSOCIAL STATUS (TRAIT ANXIETY, SELF-ESTEEM, MASTERY, DEPRESSION, SUBJECTIVE STRESS)

The next questions deal with how you have generally felt in the PAST 2 WEEKS. There are no “right” or “wrong” answers, just give the answer that best reflects HOW OFTEN you felt this way in the LAST 2 WEEKS. I will read out the possible answers a number of times and then you can just give the answer yourself.

		Almost always	Often	Sometimes	Rarely	Never
P 1	Did you feel *[during the last 2 weeks]* pleasant (2; anx)	5	4	3	2	1
P 2	In general [*during the last 2 weeks*] were you very tense and nervous (6; str)	1	2	3	4	5
P 3	Have you [*during the last 2 weeks*] felt lonely (1; dep)	1	2	3	4	5
P 4	Have you been [*during the last 2 weeks*] happy (2; anx)	5	4	3	2	1
P 5	Did you have [*during the last 2 weeks*] crying spells (1; dep)	1	2	3	4	5
P 6	Have you *[during the last 2 weeks]* felt secure (2; anx)	5	4	3	2	1

		Almost always	Ofte n	Sometime s	Rarel y	Neve r
P 7	Did you find it difficult *[during the last 2 weeks]* to 'shake off the blues', even with the help of family or friends (1; dep)	1	2	3	4	5
P 8	All in all, did you *[during the last 2 weeks]* tend to feel like a failure (4; est)	1	2	3	4	5
P 9	Have you been *[during the last 2 weeks]*, calm and collected (2, anx)	5	4	3	2	1
P 10	Did you often *[during the last 2 weeks]* have unimportant thoughts running through your mind, which bothered you (5; anx)	1	2	3	4	5
P 11	Did you feel *[during the last 2 weeks]* fearful (1; dep)	1	2	3	4	5
P 12	Did you experience disappointments *[during the last 2 weeks]* so strongly that you could not get them out of your mind (5; anx)	1	2	3	4	5
P 13	Was there *[during the last 2 weeks]* a lot of nervous strain connected to your daily activities (6, str)	1	2	3	4	5
P 14	Did you *[during the last 2 weeks]* feel hopeful about the future (2; dep)	5	4	3	2	1
P 15	Did you *[during the last 2 weeks]* feel depressed (1; dep)	1	2	3	4	5
P 16	Did you *[during the last 2 weeks]* feel sad (1; dep)	1	2	3	4	5
P 17	Were you *[during the last 2 weeks]* satisfied (content) (2; anx)	5	4	3	2	1

		Almost always	Often	Sometimes	Rarely	Never
P 18	Did you *[during the last 2 weeks]* take a positive attitude towards yourself (3; EST)	5	4	3	2	1
P 19	Did you *[during the last 2 weeks]* have a lack of self-confidence (5; anx)	1	2	3	4	5

I am now going to read a few statements. Please indicate to which extent you agree.
(Are you still OK, madam? We have come a long way already!)

		Com-pletely agree	Rather agree	I do not know	Rather disagree	Com-pletely disagree
P 20	Were you *[during the last 2 weeks]* easily bothered by things that did not use to bother you (1; dep)	1	2	3	4	5
P 21	Did you have *[during the last 2 weeks]* little control over the things happening to you (4; mst)	1	2	3	4	5
P 22	There is little you can *[during the last 2 weeks]* change about many of the important things in your life (4; mst)	1	2	3	4	5
P 23	Did you *[during the last 2 weeks]* have the feeling you do not have much to be proud of (4, est)	1	2	3	4	5
P 24	Have you *[during the last 2 weeks]* been able to do things as well as most other people (3; est)	5	4	3	2	1
P 25	Did you *[during the last 2 weeks]* often feel helpless to deal with life's problems (4; mst)	1	2	3	4	5

P 26	Did you *[during the last 2 weeks]* have the feeling that you have a number of good qualities (3; est)	5	4	3	2	1

		**Com-pletely agree**	**Rather agree**	**I do not know**	**Rather disagree**	**Com-pletely disagree**
P 27	Did you *[during the last 2 weeks]* have the feeling that you were a person of value, at least as valuable as other people (3, est)	5	4	3	2	1
P 28	Was there *[during the last 2 weeks]* really **no** way of solving some of your problems (4; mst) *Caution: question phrased negatively!*	1	2	3	4	5

General health (Stivoro, Fragerströmtest, Questions Sabine Hellemans, McFarlane)

Now we'll proceed with a number of questions about your health:

G 1	Compared to other people your age, would you say your **physical health** over the last 6 months has been…	1<br>Excellent	2<br>Very good	3<br>Good	4<br>Reasonable	5<br>Bad
G 2	Compared to other people your age, would you say your **mental health** over the last 6 months has been…	1<br>Excellent	2<br>Very good	3<br>Good	4<br>Reasonable	5<br>Bad
G 3	Have you ever smoked?	0<br>No, never smoked			1<br>Yes	

G 3a	Are you currently smoking?	0<br>No	1<br>Yes, now and again (at least 1 cigarette/week, but not daily)	2<br>Yes, at least 1 cigarette/day

G.	How long ago did you quit smoking?	1<br>1 week ago	2<br>Less than 1 month ago	3<br>Less than 6 months ago	4<br>Less than 1 year ago	5<br>More than 1 year ago

G 4	How often do you drink alcohol?	1 Never	2 Not during pregnancy	3 Occasionally, < 1/month	4 one to three x per month	5 once or twice/week	6 (almost) every day
G 5	Have you taken sleeping pills, tranquilizers, anti-depressants or anti-anxiety drugs in the last 12 months?	1 Never	2 Not during pregnancy	3 Sometimes	4 Regularly, but not anymore	5 Regularly and now as well	
G 6	Have you used drugs such as cannabis, amphetamines, ecstasy or cocaine in the last 12 months?	1 Never	2 Not during pregnancy	3 Sometimes	4 Regularly, but not anymore	5 Regularly and now as well	

We are now over halfway the interview… Is everything still OK?

(ex)partner violence (CTS2S)

The next questions deal with your relationship with your (ex)partner [in case of various (ex)partners in the last 2.5 years: I am referring to the relationship that was most difficult to you (in the past 2.5 years)]. All people in a relationship have disagreements and argue and all couples have their own way of resolving those arguments.
In the next few questions, I will list what could happen during those arguments. Please indicate HOW MANY TIMES this type of things occurred between you and your (ex)partner IN THE LAST 6 MONTHS. The figure you provide does not have to be entirely accurate. It is not easy to remember all of these things, so an approximate figure is more than sufficient.

		How often has this happened in the last 6 months?							
PG1	You explained your side, or suggested a compromise, in an argument with your (ex)partner	1 **1 x**	2 **2 x**	3 **3 -5 x**	4 **6 - 10 x**	5 **11 - 20 x**	6 **> 20 x**	7 **Not in the last 6 months, but before**	8 **Never**
PG2	Your (ex)partner clarified his/her side, or suggested a compromise, in an argument with you	1 **1 x**	2 **2 x**	3 **3 -5 x**	4 **6 - 10 x**	5 **11 - 20 x**	6 **> 20 x**	7 **Not in the last 6 months, but before**	8 **Never**
PG3	You insulted your (ex)partner, or swore, shouted or screamed at him/her	1 **1 x**	2 **2 x**	3 **3 -5 x**	4 **6 - 10 x**	5 **11 - 20 x**	6 **> 20 x**	7 **Not in the last 6 months,**	8 **Never**

								**but before**	

How often has this happened in the last 6 months?									
PG4	Your (ex)partner insulted you, or swore, shouted or screamed at you	1 **1 x**	2 **2 x**	3 **3 -5 x**	4 **6 - 10 x**	5 **11 - 20 x**	6 **> 20 x**	7 **Not in the last 6 months, but before**	8 **Never**
PG5	You had a sprain, bruise or small cut or felt pain the next day after a fight with your (ex)partner	1 **1 x**	2 **2 x**	3 **3 -5 x**	4 **6 - 10 x**	5 **11 - 20 x**	6 **> 20 x**	7 **Not in the last 6 months, but before**	8 **Never**
PG6	Your (ex)partner had sustained a sprain, bruise or small cut or felt pain the next day after a fight with you	1 **1 x**	2 **2 x**	3 **3 -5 x**	4 **6 - 10 x**	5 **11 - 20 x**	6 **> 20 x**	7 **Not in the last 6 months, but before**	8 **Never**
PG7	You showed respect for, or showed that you cared for your (ex)partner's feelings about an issue you disagreed on	1 **1 x**	2 **2 x**	3 **3 -5 x**	4 **6 - 10 x**	5 **11 - 20 x**	6 **> 20 x**	7 **Not in the last 6 months, but before**	8 **Never**
PG8	Your (ex)partner showed respect for, or showed that he/she cared for your feelings about an issue you disagreed on	1 **1 x**	2 **2 x**	3 **3 -5 x**	4 **6 - 10 x**	5 **11 - 20 x**	6 **> 20 x**	7 **Not in the last 6 months, but before**	8 **Never**
PG9	You pushed, jabbed or slapped your (ex)partner	1 **1 x**	2 **2 x**	3 **3 -5 x**	4 **6 - 10 x**	5 **11 - 20 x**	6 **> 20 x**	7 **Not in the last 6 months, but before**	8 **Never**
PG 10	Your (ex)partner pushed, jabbed or slapped you	1 **1 x**	2 **2 x**	3 **3 -5 x**	4 **6 - 10 x**	5 **11 - 20 x**	6 **> 20 x**	7 **Not in the last 6 months, but before**	8 **Never**

		How often has this happened in the last 6 months?							
PG 11	You punched or kicked your (ex)partner or you beat him/her up	1 **1 x**	2 **2 x**	3 **3 -5 x**	4 **6 - 10 x**	5 **11 - 20 x**	6 **> 20 x**	7 **Not in the last 6 months, but before**	8 **Never**
PG 12	Your (ex)partner punched or kicked you (ex)partner or beat you up	1 **1 x**	2 **2 x**	3 **3 -5 x**	4 **6 - 10 x**	5 **11 - 20 x**	6 **> 20 x**	7 **Not in the last 6 months, but before**	8 **Never**
PG 13	You destroyed something belonging to your (ex)partner or threatened to hit him/her	1 **1 x**	2 **2 x**	3 **3 -5 x**	4 **6 - 10 x**	5 **11 - 20 x**	6 **> 20 x**	7 **Not in the last 6 months, but before**	8 **Never**
PG 14	Your (ex)partner destroyed something belonging to you or threatened to hit you	1 **1 x**	2 **2 x**	3 **3 -5 x**	4 **6 - 10 x**	5 **11 - 20 x**	6 **> 20 x**	7 **Not in the last 6 months, but before**	8 **Never**
PG 15	You went to a doctor or needed to see a doctor because you had been in a fight with your (ex)partner	1 **1 x**	2 **2 x**	3 **3 -5 x**	4 **6 - 10 x**	5 **11 - 20 x**	6 **> 20 x**	7 **Not in the last 6 months, but before**	8 **Never**
PG 16	Your (ex)partner went to a doctor because or needed to see a doctor he/she had been in a fight with you	1 **1 x**	2 **2 x**	3 **3 -5 x**	4 **6 - 10 x**	5 **11 - 20 x**	6 **> 20 x**	7 **Not in the last 6 months, but before**	8 **Never**
PG 17	You used force (like hitting, holding down or using a weapon) to make your (ex)partner have sex with you	1 **1 x**	2 **2 x**	3 **3 -5 x**	4 **6 - 10 x**	5 **11 - 20 x**	6 **> 20 x**	7 **Not in the last 6 months, but before**	8 **Never**

How often has this happened in the last 6 months?									
PG 18	Your (ex)partner used force (like hitting, holding down or using a weapon) to make you have sex with him/her	1 **1 x**	2 **2 x**	3 **3 -5 x**	4 **6 - 10 x**	5 **11 - 20 x**	6 **> 20 x**	7 **Not in the last 6 months, but before**	8 **Never**
PG 19	You insisted on sex when your (ex)partner did not want to / or insisted that you had sex without a condom (without using physical force)	1 **1 x**	2 **2 x**	3 **3 -5 x**	4 **6 - 10 x**	5 **11 - 20 x**	6 **> 20 x**	7 **Not in the last 6 months, but before**	8 **Never**
PG 20	Your (ex)partner insisted on sex when you did not want to / or insisted that you had sex without a condom (without using physical force)	1 **1 x**	2 **2 x**	3 **3 -5 x**	4 **6 - 10 x**	5 **11 - 20 x**	6 **> 20 x**	7 **Not in the last 6 months, but before**	8 **Never**

Readiness to change (Hegarty et al., 2008; Frasier et al., 2001)

RTC 1	Have you thought about making any changes to your current situation (concerning your relationship) within the **next 6 months**?	0 No	1 Yes
RTC 2	Have you thought about making any changes [to your current situation concerning your relationship] within the **next 30 days**?	0 No	1 Yes

Are you still OK? We have come a long way, we need max 5 minutes.

Safety promoting behaviour (Safety promoting behavior checklist, McFarlane et al., 2004)

Sometimes, arguments get out of hand and you need to do certain things to feel safer. That is what the following questions are about.

VG 1	Have you (in the past 6 months) made sure that you had a small amount of money on you to make a call, take the bus or taxi in case of emergency?	0 No	1 Yes

VG 2	Have you (in the past 6 months) prepared an emergency bag with clothes, spare house and/or car keys, copies of important documents (social security card, birth certificate, driving license, ID card, bank card …) and stored it in a safe location	0 No	1 Yes
VG 3	Have you (in the last 6 months) agreed on a code (e.g. switching lights on and off) with neighbours, relatives or someone else so they know that they have to call the police when you need help.	0 No	1 Yes
VG 4	Have you (in the last 6 months) removed objects that could be used as a weapon	0 No	1 Yes

It may seem odd that I am asking these questions, but we advise you to do these things if you do not feel safe, of course you are not obliged to do this…

Help-seeking behaviour (Community agencies use, questionnaire McFarlane et al. 2006; Fanslow et al., 2010 p 939)

The following questions are about the help you sought for your problems with your (ex)partner. Have you, in the last 6 months, because of your problems with your (ex)partner, contacted one or more of the following services?

If so, how many times and how helpful did you find these services/this service to deal your problems with your (ex)partner?

1= very helpful, 2= somewhat helpful, 3= not helpful, 4= made things worse

	**Service**	**No**	**Yes**	**Number of times**	**To which extent were you helped**
HG1	Centrum voor Algemeen Welzijnswerk (CAW)	0↓	1→		1 2 3 4
HG2	Centrum geestelijke gezondheidszorg (Mental Health Centre)	0↓	1→		1 2 3 4
HG3	Vluchthuis (Women's shelter, safe house)	0↓	1→		1 2 3 4
HG4	Hospital Social Services	0↓	1→		1 2 3 4
HG5	Tele-onthaal (telephone hotline) Over-the-phone assistance	0↓	1→		1 2 3 4

HG6	Self-help group	0↓	1→		1 2 3 4
	**Service**	**No**	**Yes**	**Number of times**	**To which extent were you helped**
HG7	Police	0↓	1→		1 2 3 4
HG8	Legal services (legal aid, lawyer, court, …)	0↓	1→		1 2 3 4
HG9	Other: …………………….	0↓	1→		1 2 3 4
HG10	**If YES to one item HB 1 -9:**<br>**What was the immediate cause/reason to contact that service/those services? I will read out a number of possible reasons:**<br>☐ 1 I could not take it anymore<br>☐ 2 (Ex) (ex)partner threatened or tried to kill me<br>☐ 3 Severely injured by (ex)partner<br>☐ 4 Afraid that (ex)partner would kill me<br>☐ 5 Children are suffering because of the situation<br>☐ 6 Children were beaten or threatened<br>☐ 7 Under the influence of family and/or friends<br>☐ 8 Pregnancy/baby<br>☐ 9 Other: …………………………………………………………………..				
HG11	**If NO to all items HB 1 - 9 :**<br>**Why did you not contact one or more of these services? I will read out a number of possible reasons:**<br>☐ 1 the situation/(ex)partner violence is normal or not serious (enough)<br>☐ 2 I am ashamed or embarrassed about the (ex)partner violence				

	☐ 3 I am afraid of the consequences<br>☐ 4 I need to solve this myself/alone<br>☐ 5 I am afraid of losing the children<br>☐ 6 Those services cannot help me anyway<br>☐ 7 Other: …………………………………………………………………..		
HG 12	Did you, (outside of the services just mentioned), talk to someone about your problems with your (ex)partner?	0<br>No	1<br>Yes
HG 13	**IF SO**, to whom?<br>☐ 1 Family<br>☐ 2 Friend (male or female)<br>☐ 3 Acquaintance<br>☐ 4 Neighbour<br>☐ 5 Co-worker<br>☐ 6 Family doctor<br>☐ 7 Gynaecologist<br>☐ 8 Midwife<br>☐ 9 ER doctor<br>☐ 10 ER nurse<br>☐ 11 Psychologist<br>☐ 12 Psychiatrist<br>☐ 13 Social worker<br>☐ 14 Other: …………………….		

Social support (questionnaire Wendy K&G and/or BIDENS question)

Well, this brings us almost to the last questions of the interview.

SS 1	Do you have someone, apart from your (ex)partner, to really confide in?	0<br>No	1<br>Yes, 1 or 2 persons	2<br>Yes, >2 persons

If you need to, who could you turn to for each of the following things?

		No-one	(ex)partner	Parents	Brothers/ Sisters	Parents in-law	Neighbours	Co-workers	Friends	Others specify
SS 2	Talking about personal problems or intimate matters	1	2	3	4	5	6	7	8	9
SS 3	Advice and counsel	1	2	3	4	5	6	7	8	9
SS 4	Help with practical matters (odd jobs, transport, housekeeping)	1	2	3	4	5	6	7	8	9

SS 5	Financial support	1	2	3	4	5	6	7	8	9
SS 6	Having fun or relaxing together	1	2	3	4	5	6	7	8	9
SS 7	Having a nice chat	1	2	3	4	5	6	7	8	9

Controle interventie

1= very helpful, 2= somewhat helpful, 3= not helpful, 4= made things worse

I 1	During your consultation after your delivery, did you receive an envelope regarding this study, containing a plastic card and gift voucher?	0 No	1 Yes	2 I do not remember	
I 2	To which extent did this **card** help you to deal with the problems involving your (ex) partner?	1	2	3	4
I 3	To which extent did filling in the **questionnaire** help you to deal with the problems involving your (ex) partner?	1	2	3	4
I 4	To which extent did this **interview** help you to deal with the problems involving your (ex) partner?	1	2	3	4

Conclude

As a last point, I would like to know what your gynaecologist or midwife could do to help you with your problems with your (ex)partner? What would have helped you during your pregnancy?

………
………
………
………
………
………
………
………
………
………
………
………
………
………
………

……
……
……
……
………

Are there any other important things you would like to tell me?

……
……
……
……
……
……
……
……
……
……
……
……
……
……
……
……
……
……

Do you need to talk to a professional care worker?
(CAW 078/150 300, www.caw.be, tele-onthaal 106/107)

CAW1	Gave number	No<br>0	Yes<br>1

Well, this concludes this first interview.
If you remember, the study consists of 2 interviews. In other words, we will call you again in 6 months to do the follow-up interview

Again, thank you very much for your time and participation in the study.

Respondent in **intervention group** (referral card):
Respondent in **control group** (thank you card):
